# 

**Published:** 1875-01

**Authors:** 


### TABLE OF CONTENTS.

ORIGINAL COMMUNICATIONS.

Hypodermic Medication. By J. B. Mattison, M.D. : : : : : 1
Tincturæ Nucis Vomicæ an Antiperiodic. By. W. W. Fierce, M.D. : : 7
Bromide of Potassium. By W. F. Barr, M.D. : : : : : : 11
Clay as a Therapeutic Agent. By A. L. Knight, M.D. : : : : 14
Gun-Shot Wound—Tetanus—Chloral. By A. R. Dilpatrick, M.D. : : 18
A Case of Strangulated Hernia. By J. W. Wimbish, M.D. : : : 20
Atropia in Phthisical Sweating. By A. G. Smythe, M.D. : : : 21

SELECTIONS.

A Case of Membranous Dysmenorrhœa. : : : : : : 22
The Injection of Pulmonary Cavities. : : : : : : : 25
Hot Water Injections in Uterine Diseases. : : : : : : 26
Application of the Forceps. : : : : : : : : : 28

EXTRACTS AND GLEANINGS.

Principal Cause of Constitutional Derangement in First Dentition; Infant Diet; Diarrhœa of Typhoid Fever; The Claims of the Medical Profession; Muriate of Ammonia for Nervous Diseases; Points in the Treatment of Diseases of Children; Acetic Acid in Mucous Polypus; A New Method for Healing Ulcers; Hydrate of Chloral as a Solvent; For Incontinence of Urine in Paralysis; Beneficial Effects of Vaccination; Keeping the Bed after Confinement; Diptheria; The Treatment of Phthisis; Nicotine as an Antidote to Strychnine; Iodoform in Granular Lids; Gurjun Oil in Leprosy; "Persistent" Inflammation of Joints; Typhoid Fever; Erysipelas; Lacto-Phosphate of Lime in Mineral Inanition; Why do we give Wine in Fever?; Iodic Acid in Hypodermic Injections; Cardiac Disease; Hiccough and Spasmodic Affections—Oil of Amber; Black Cohosh in Rheumatism; Puerperal Mania—Chloral Hydrate; Remedy for Chronic Hoarseness; Positions for Catheterism; For Uterine Neuralgia; Formnla for Intertrigo; The Administration of Phosphorous; Compound Syrup for Garlic; Pyrosis; Causation of Typhoid or Enteric Fever; Delirium in Typhoid Fever; Chloroform in Strychnine Poisoning; Digitalis; Eczema Capitis; External Use of Turpentine in the Treatment of Tonsilitis; Ulceration of the Vagina, Larynx, and other parts, in Typhoid Fever; Oat Meal Farina as a Food for Infants; Anointing with Cocoa Butter in Scarlet Fever; Prolapsed Rectum; New Antiseptic Ointment; Carbolic Acid in Tapeworm; Hypodermic Injection of Morphia and Atropine; Diphtheria; Formulas for Diarrhœa and Dysentery in Children; Lentil Meal; Solution of Morphia for Hypodermic Injection; Mammary Abcess (Threatened)—Application of Oleate of Mercury; Use of Gelatin Suppositories in Obstinate Constipation due to Accumulation of Fæces in the Rectum; Fatal Poisoning from Chlorate of Potassa; Resemblance of Twins; Hooping-Cough; Catarrh; To Check Vomiting from Cough; Erysipelas—Styptic Colloid; Chloral and Ipecacuanha in Croup; Delirlum Tremens after Chloral-Drinking; Enlargement of the Tonsils as a Cause of Nightmare; Cholera; Death from the use of Perchloride of Iron. : 30

EDITORIAL AND MISCELLANEOUS.

To our Subscribers. : : : : : : : : : : 58
Glad Greetings. : : : : : : : : : : : 59
To Our Exchanges. : : : : : : : : : : . 60
Communications. : : : : : : : : : : : 61
University Publishing Company—New York and Baltimore. : : : 61
Notice of Postponement. : : : : : : : : : 61
Reviews. : : : : : : : : : : : : : 62
Vick's Floral Guide. : : : : : : : : : 64
St. Nicholas. : : : : : : : : : : 64
List of Cash Payments to the Record for 1875. : : : : : : 54

